# Topographic Features of Five K-file Brands in Iranian Market: A Scanning Electron Microscopic Study 

**DOI:** 10.22037/iej.v12i3.16031

**Published:** 2018

**Authors:** Arash Shahravan, Hedayat Gorjestani, Arash Izadi, Nazanin Mortazavi

**Affiliations:** a *Endodontology Research Center, Kerman University of Medical Sciences, Kerman, Iran; *; b *Endodontics, Private Practice, Vancouver, British Columbia, Canada; *; c * Dental Research Center, Department of Endodontics, Dental School, Golestan University of Medical Sciences, Gorgan, Iran; *; d * Dental Research Center, Department of Oral and Maxillofacial Medicine, Dental School, Golestan University of Medical Sciences, Gorgan, Iran*

**Keywords:** Endodontic K-files, Scanning Electron Microscopy, Topography

## Abstract

**Introduction:**

Endodontic files which are used to clean and shape the root canal space differ from each other regarding technical specifications. Recently, K-type files are repeatedly studied on their cutting efficiency. This study aims to evaluate the tip design and cutting efficiency of 5 brands of K-files, available in Iran dental market (naming Dentsply, Thomas, Mani, Perfect and Larmrose).

**Methods and Materials:**

In this descriptive study, topographic features of file tips were investigated by the scanning electron microscope (SEM). Those features included tip symmetry, tip design, tip angle, and the distance from the tip to the lowest flute. SEM images (×250 magnification) of files were prepared. Statistical tests (Fisher's exact test, *Chi*-square, ANOVA, and *t* test) were used and *P*<0.05 was considered as significant.

**Results:**

Dentsply files had the most number of morphologically pyramidal sharp tips and the greatest tip angles. However, Larmrose files were the most frequent files having cutting sharp tips. Symmetrical tips existed among 100% of Dentsply and Mani brands. No significant differences were found with respect to distance from the file tip to the lowermost flute between different file brands of this study (*P*=0.2, One way ANOVA).

**Conclusion:**

Dentsply and Mani files possessed the most symmetrical tips and greatest tip angles. With respect to tip length, all 5 brands were satisfactory. However, neither of 5 brands evaluated topographically were outstanding in every aspect.

## Introduction

Clinicians cannot make informed choices between endodontic instruments unless they are told about details of related research [1]. Hence, research on developing endodontic technology and testing the continually changing instrumentation materials is crucial [2]. In this context, providers of treatment need more information from manufacturers regarding machining and cutting of endodontic instruments [3]. The wide variations in the diameters and tapers of nominally the same size endodontic instruments prompted the international standard organization (ISO) and dental supply houses to consider standardization of such instruments [4, 5]. 

Currently, endodontic treatment involves removal of the irreversibly damaged pulp, followed by cleaning and shaping of the root canal space and subsequent obturation [6, 7]. Endodontic treatment has a good prognosis if root canals are instrumented to the physiological apex [8].

The ideal objective of instrumentation is to clean and shape canals with minimal dentin removal. This objective is not always attainable, especially in curved canals [9]. Instruments such as rigid files tend to straighten curved canals resulting in ledge formation and apical transportation [10]. File tip modification provides better control of the canal preparation size and produces smooth preparations as opposed to varying degrees of ledging by non-modified hand instruments [11]. Dell Bello *et al.* [12] in 1988 proposed combining the crown-down technique with an endodontic file possessing tip guidance and reported less canal aberration and instrument breakage by this combination.

The use of a modified double-flared technique with non-cutting tip files was introduced in 1992 to prepare curved root canals [13]. In 2012-2014, considerable improvement in the design and raw material of nickel-titanium rotary endodontic files has been reported [14]. In 2017, however, a low incidence of fracture was found when reciprocating files were used in conjunction with the traditional K-type files in few cases of endodontic treatment [15]. Technological advances in the design and manufacturing process of endodontic files have dramatically changed the profile of non-surgical root canal therapy. However, differences in the tip design, cross sectional shapes and helical angles correspond to manufacturers’ standards [16]. 

More recently, the impact of physical properties of K-files on their cutting efficiency has been focused [17, 18]. The aim of the present study is to evaluate the topographic features of 5 common brands of K-files in Iranian dental market. Tip design study includes tip morphology, tip angle, tip symmetry and the distance from the tip to the lowest flute.

## Materials and Methods

The following brands of K-files as more common bands in Iranian dental market were selected: Dentsply (Dentsply 

Maillerfer, Ballaigues, Switzerland), Thomas (French Dental Products, Société-FFDM Pneumat, Département Dentaire Thomas, Bourges Cedex, France), Mani (Mani, Tochigi, Japan), Perfect (Shenzhen, Guangdong, China) and Larmrose (Taizhou, China, Beijing). Then, from each brand, six files from each size (# 15 to # 30) were selected (*n*=24).

***Image preparation***

To investigate topographic features of endodontic files, a scanning electron microscope (SEM), Cam Scan MV2300 (Cam Scan, Cambridgeshire, UK) was applied. SEM micrographs were taken from file tip region under 250× magnification and 1 kVp voltage. For this purpose a rapid-setting glue (cyanoacrylate) was rubbed on discs of the SEM, to which main parts of files were attached (plastic handles removed, before). Then, other non-gluey sides were photographed. The prepared micrographs were saved on computer as JPEG formats. Arranged facts and figures were evaluated by two observers who were not aware of the studied file brands. Where contradictions existed, a third person was called for. 

**Table 1:** File distribution on the basis of tip morphology

**Brand**	**Pyramidal with sharp tip**	**Pyramidal with blunt tip**	**Sharp tip**
**Perfect**	3 (12.5 %)	20 (83.3%)	1 (4.16%)
**Larmrose**	6 (25%)	6 (25%)	12 (50%)
**Mani**	6 (25%)	18 (75%)	0
**Thomas**	6 (25%)	11 (45.8%)	7 (29.16%)
**Dentply**	9 (37.5 %)	15 (62.5%)	0

**Table 2:** File tip angles and distances from tip to the lowest flute

**Brand**	**Mean of tip angles (Degree)**	**Distance from tip to the lowest flute** **(µm)**
**Perfect**	57.65 (6.2)	135.35
**Larmrose**	55.25 (5.2)	195.61
**Mani**	74.25 (7.1)	131.22
**Thomas**	51.5 (4.8)	186.25
**Dentsply**	73 (6.9)	132.1

**Table 3:** The number (percent) of files with sharp / blunt tips

	**Dentsply**		**Mani**		**Thomas**		**Larmrose**		**Perfect**	
**Size**	**Sharp**	**Blunt**	**Sharp**	**Blunt**	**Sharp**	**Blunt**	**Sharp**	**Blunt**	**Sharp**	**Blunt**
**15**	3 (0.3)	3 (0.3)	3 (0.3)	3 (0.3)	4 (0.4)	2 (0.2)	2 (0.2)	4 (0.4)	1 (0.1)	5 (0.5)
**20**	3 (0.3)	3 (0.3)	0	6 (0.6)	1 (0.1)	5 (0.5)	5 (0.5)	1 (0.1)	0	6 (0.6)
**25**	1 (0.1)	5 (0.5)	1 (0.1)	5 (0.5)	2 (0.2)	4 (0.4)	5 (0.5)	1 (0.1)	2 (0.2)	4 (0.4)
**30**	2 (0.2)	4 (0.4)	2 (0.2)	4 (0.4)	6 (0.6)	0	6 (0.6)	0	0	6 (0.6)
**Total**	9 (1)	15(1.6)	6 (0.6)	18 (2)	13(1.4)	11(1.2)	18^* ^(2)	6 (0.6)	3(0.3)	21(2.3)

**Figure 1:**
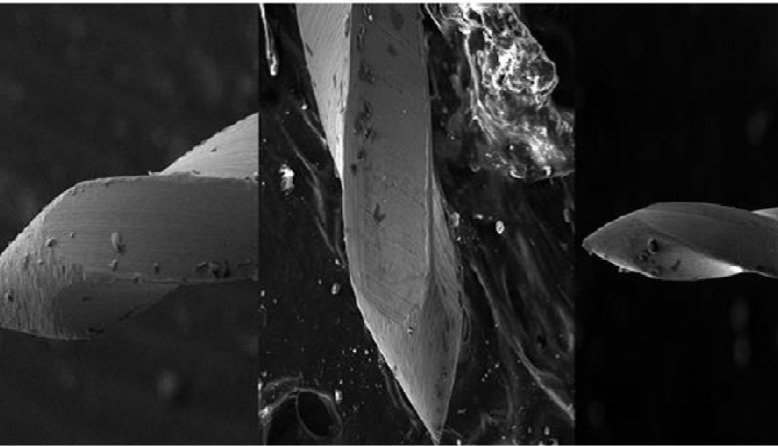
Three types of studied file tip (right to left): pyramidal with sharp tip, sharp angle, and blunt tip

***Topographic*** ***features***

**Figure 2:**
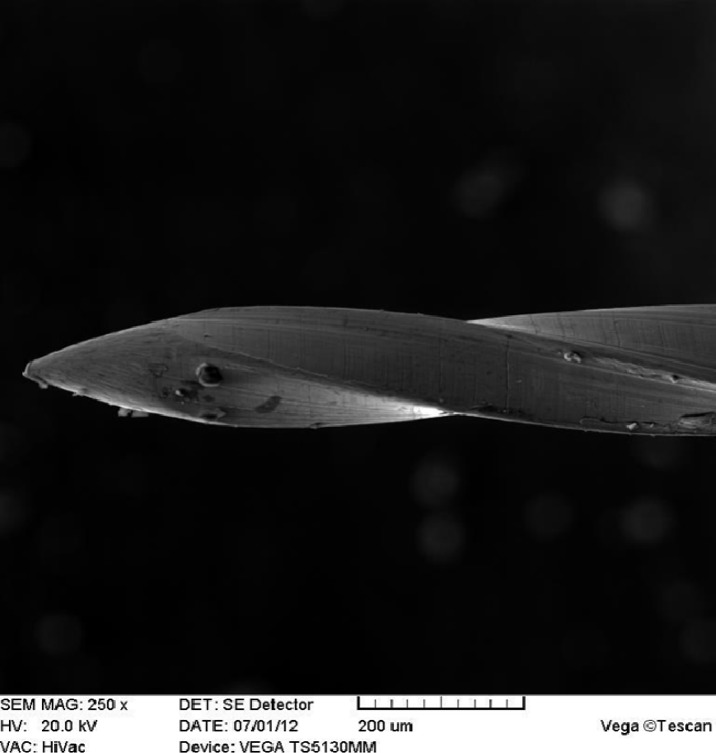
SEM image of a file tip (Larmrose #25)

Three file tip design were defined as pyramidal with blunt tip, pyramidal with sharp tip, and sharp angle tip (Figures 1 and 2).

**Figure 3:**
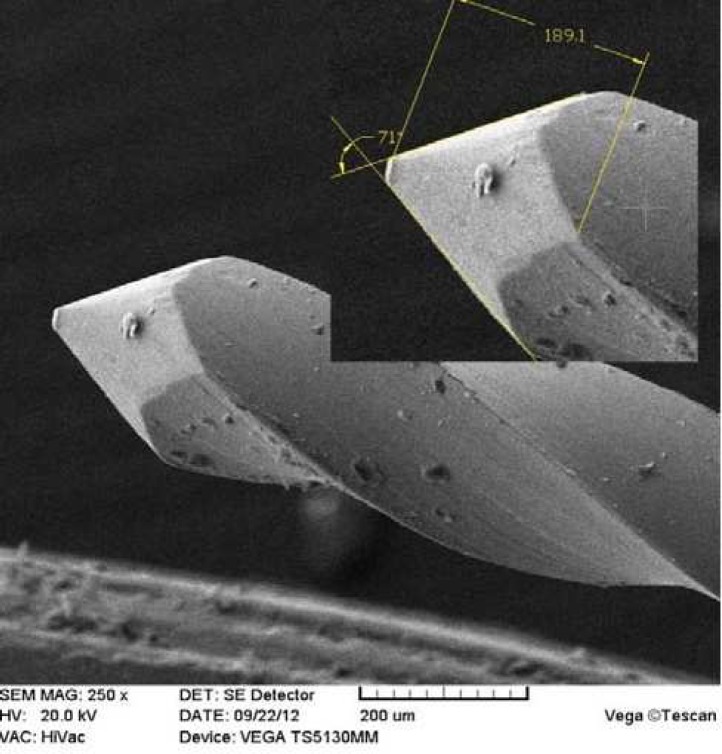
File tip angle and the distance from the lowest flute to the tip

File tip angle and the distance from the lowest flute to tip SEM images from different files were saved as Photoshop CS4 formats, then tip angles and distances from the lowest flutes to tips were separately measured (Figure 3).

File tip symmetry was evaluated using the Adobe Photoshop CS5. For this purpose, longitudinal lines were drawn from both sides of file toward its tip.

Cutting was numerically determined for each file according to its tip sharpness (Table 3). A line parallel to each file side was drawn. Then, the external angle at the crossings was labeled as the numerical value of the file cutting efficiency. Statistical tests (Fisher's exact test, *Chi* square, ANOVA, and *t* test) were used and *P*<0.05 was considered as significant.

## Results

***Tip symmetry:*** All 18 files from Perfect (75%), 20 files from Thomas (83.3 %), 22 files from Larmrose (91.7%), and all 24 files from Mani and Dentsply (100%) had symmetrical tips. Statistically significant differences existed among those frequencies of different brands (*P*=0.014, X^2^=12.59).

***Tip design:*** Table 1 demonstrates file tip shapes in relation to the size and brand. Accordingly, 9 files from Dentsply (37.5%) had pyramidal sharp tips and other brands had less. Statistically significant differences existed regarding the tip design between different brands (*P*<0.0001); Dentsply, Thomas, Mani and Perfect had the most pyramidal blunt tips while Larmrose had the most (50%) sharp tips. 

***Tip angle*** ***and the distance from tip to the lowest flute:*** SEM images were evaluated through software Photoshop CS5. Table 2 demonstrates studied files tip angles as well as distances from the tip to the lowest flute. The latter showed no statistically significant differences among different file brands (*P*=0.2)

***Cutting:*** for this criteria 5 file brands were studied with respect to their tip cutting or sharpness (Table 3). Larmrose had the most number of cutting tips and perfect had the least. Other brands had intermediary cutting properties (X^2^=23.93, *P*=0.001). Table 3, quantitatively, demonstrates the cutting or non-cutting number of files in different brands and sizes.

## Discussion

The present study has applied SEM images for the evaluation of endodontic files. The history of SEM images for studying file topography backs to 1980s, where Comier *et al.* [4] found significant differences between file topography of 6 different brands. Stereo-microscopic views have already been used for the physical studies of endodontic files such as flute numbers [19]. However, SEM images are more delicate than the latter and file topography is exclusively studied by SEM [20, 21]. There is a direct relationship between the file tip’s shape and cutting efficiency of the file, that is, files with sharp tips have more cutting efficiency than blunt files. The sharp and cutting tips remove more debris (and dentin) during canal preparation. However, this aggressive tissue removal may increase the risk of apical transportation and perforation [22, 23]. In our study, Perfect^® ^included the least number of sharply-tipped files and Larmrose^® ^included the most. Other file brands had intermediary properties. 

File tip morphology is another instrument characteristic that affects shaping and cleaning of the root canal space [24]. Miserendino *et al.* [24] have defined three types of file tips: sharp tip, pyramydal with sharp tip and pyramidal with blunt tip. Their definition was used for our evaluation of files. While tissue removal by files with pyramidal-blunt tip requires more intra-canal function of file, this may have the advantage of decreasing procedural risks [24, 25]. The present study, however, did not evaluate the extent of tissue removal.

Newman *et al.* [22] have already demonstrated that with an increase in the number of flutes, the cutting of files will also increase. In our previous study, Larmrose files had the most number of flutes [26]. It may be estimated that Larmrose files having most number of sharp tips and flutes remove more tissues than other files. However, additional investigation is necessary in this regard. Previous studies have revealed that the file will have the most cutting if its tip length (the distance from the tip to the lowest flute) is less than 1 mm [25]. Our experimental files at the present trial had tip lengths of less than 1 mm; an indication for proper cutting efficiency of all 5 studied brands.

In the present study, only Larmrose and Perfect files had tip angles approaching the 60 degrees (Table 2). Miserendino *et al.* [24] have demonstrated that file tip design affects tissue removal by files. According to these investigators, tip angles (60-69 degrees) at narrow canals and tip angles (40-49 degrees) at wider canals have greater cutting efficiency. If this is true, our experimental files may not have ideal cutting. However, those file brands should have proper cutting efficiency with respect to their tip lengths of less than 1 mm (Table 2). 

There were certain limitations on what we could distinguish a real brand file from a fake one. Even the dental supply house does not seem able to distinguish between real and fake files. We recommend extensive research on existing new files in Iran dental market to find out more information about them. In this case, dentists can have the first choice of files.

## Conclusion

Dentsply and Mani files possessed the most symmetrical tips and greatest tip angles. However, the most frequent cutting tips belong to Larmrose files. The results of the present study indicated that none of the tested file brands were outstanding in every aspect.
